# Environment in Children’s Health: A New Challenge for Risk Assessment

**DOI:** 10.3390/ijerph181910445

**Published:** 2021-10-04

**Authors:** Francesca Mastorci, Nunzia Linzalone, Lamia Ait-Ali, Alessandro Pingitore

**Affiliations:** Biocomplexity Lab, Clinical Physiology Institute, National Research Council (NRC), Via Moruzzi 1, 56124 Pisa, PI, Italy; mastorcif@ifc.cnr.it (F.M.); linunzia@ifc.cnr.it (N.L.); aitlamia@ifc.cnr.it (L.A.-A.)

**Keywords:** environmental contaminants, children, health, fetal, perinatal period, risk assessment

## Abstract

In the last few years, many studies have focused on the effects of environmental contaminant exposure during the prenatal period or infancy as predictors of health outcomes in the future. In these time windows, due to their rapid growth, and physiologic and metabolic development, we can observe a higher vulnerability to the effects of environment, with respect to adulthood. The evidence of possible influences, partly mediated by epigenetic mechanisms, involve neurobehavioral responses and immune, endocrine, and respiratory systems, acting directly on the child or indirectly when mediated by placental transfer or breast feeding. In particular, due to a greater intake of air, food, and fluids relative to body weight, crawling behaviors and short stature, the risk of excessive exposure is greater in children. However, data on the long-term implications of early exposures are scarce. Additionally, so that physicians and institutions for child care and assistance of pregnant women can take actions to counteract the effects of chemical pollution (i.e., by educational opportunities), a risk assessment perspective that responds to the biocomplexity of the human being is needed. The present paper provides an overview of physiologic and behavioral characteristics during the perinatal period and in childhood, suggesting in a more integrated way, the need of a new risk-assessment approach to managing chronic disease in pediatric patients.

## 1. Introduction

Protection and prevention of health against disease caused by environmental contaminants are the ultimate goals in the fields of human risk assessment and risk management. Usually, these areas of intervention are focused on exposures in adulthood, with the development and implementation of more innovative methodologies for estimating risks [1]; on the contrary, little attention is given to embryonic and fetal periods and childhood, although many diseases later in life caused by toxic agents find their onset in these time windows. In this regard, it is not correct to consider children or adolescents as little adults, but rather as a sensitive target population where biological systems are developing and redefining [2]. In particular, the metabolic processes of absorption and the elimination of environmental contaminants in children are slower than in adults, making them more susceptible to disease outcomes following even small doses [3]. In addition, children’s daily activities of crawling increases hand-to-mouth ingestion, making them vulnerable to greater contact with certain chemicals.

Based on animal and human studies, during different periods of growth, the consequences of exposure to toxicant chemicals have the potential to alter anatomic structures and physiological functions, with costs for endocrine, neurological, sexual and behavioral development over the life span. Therefore, from a public health and preventive medicine point of view, the perinatal period and childhood require a risk assessment that takes into account specific characteristics, highlighting factors for a child-protection focused on data from contact with pesticides and several potential harmful contaminants, such as lead, arsenic, and particulate matter, especially in the long-term perspective. Currently, the total environment model is a new frontier in children’s environmental health research [4]. 

Despite the wealth of literature on the effects of environmental contaminant exposure on adults, there is less information regarding these effects during pregnancy or in childhood as predictors of health outcomes in the future. In this review, we attempt to move towards this goal. To our knowledge, no data have been reported so far. Rather, we present an overview of physiologic and behavioral characteristics during the perinatal period and in childhood, with the aim of suggesting, in a more integrated way, a new framework for risk assessment approach, particularly across the early stages of life. 

## 2. Vulnerability in Perinatal Period and Infancy

The metabolism and the ability of children to excrete contaminants is very different from adults; in some cases showing great vulnerability [5,6]. Differences exist also during the perinatal period, in particular between prenatal and postnatal life, mainly because of the not-yet-fully-developed blood–brain barrier, which allows the passage of xenobiotics to the central nervous system [7]. The perinatal period, due to quick physiological changes, is a crucial phase for understanding how and what exposure to environmental contaminants can induce long-term impact on neurodevelopment and behavior. In particular, embryonic, fetal life, and infancy represent critical sensitive windows of susceptibility in which the primary structures of the central nervous system (CNS) are forming and organizing. In addition, the enzymes responsible for detoxifying processes have not yet optimized their metabolic abilities [8]. Furthermore, placental transfer, breast milk, or inhalation, ingestions, and physical contact with different food and objects can augment the exposure to various toxic agents.

In the neurodevelopment phase, different events from internal and external environments send signals in order to control complex physiological processes [9,10]. This evolutionary plasticity, on the one hand, is necessary for the proper development of the brain; however, on the other hand, it leaves the brain more vulnerable to internal and external stimuli [11]. For this reason, exposure to environmental contaminants during this sensitive window can induce permanent or long-term alterations to the brain and other organs, with effects on physiological mechanisms and behavioral dynamics. Among the different possible long-term effects, impairments in the development of the brain, disabilities in learning, memory, and emotional responses have been described [12].

During embryonic and fetal stages, the development of endocrine systems, especially those related to reproduction functions in adults, can be compromised by endocrine-disrupting chemicals (EDCs), with consequent neurological and reproductive dysfunction [13]. Both animal and human studies have revealed that during pregnancy and breastfeeding, mothers and, consequently, their children, are exposed to endocrine-disrupting chemicals and toxic metals present in foodstuffs, which, accumulating in the placenta, can cause preeclampsia, increased uterine vascular resistance, impaired placental vascularization, and increased gestational diabetes in the mother, resulting in intrauterine growth restriction and preterm birth in the child [14]. EDs are chemicals that disrupt typical endocrine functions and include synthetic organic compounds, such as pharmaceutical agents, plant protection products, plastics, plasticizers, polychlorinated biphenyls, dioxins, flame-retardants, and antifoulant paint additive, as well as natural substances derived from plants [15,16,17]. Children, directly or indirectly, are daily exposed to EDCs by drinking contaminated water, eating, breathing polluted air or by direct contact with chemicals, disrupting female reproductive tract development, testosterone synthesis and sexual differentiation, leading to adult testis dysfunction, and infertility [18]. In particular, many animal and human studies have revealed that EDs during embryonic and fetal periods induce harmful effects, not only on exposed subjects, but also in future generations, according to epigenetic mechanisms that refer to a transgenerational inheritance [19], suggesting the need of a new approach to noncommunicable diseases that consider the evolutionary origins of disease. In fact, many neurodevelopmental disabilities, such as autism, attention-deficit/hyperactivity disorder (ADHD), dyslexia, or behavioral disorders, seem to be related to toxic chemicals that interfere with normal fetal neurodevelopment [20].

Until a few years ago, evidence of the negative effects of maternal exposure to EDCs during gestation and/or lactation came from only animal research, documenting long-term alterations in offspring behavior [21,22]. More recently, emerging human studies on maternal bisphenol A (BPA) provide data on the increase in non-communicable diseases, such as endometriosis, infertility, obesity, diabetes, early puberty, susceptibility to infections, autoimmune diseases, learning disabilities, neurodegenerative diseases, and heart disease [23]. The great attention to bisphenol is linked to the fact that about 95% of the human population reports measurable levels of BPA in their bodies, including in newborn babies with a high level in plasma, liver, and amniotic fluid [24]. However, the involvement of BPA in compromising human health remains controversial. Despite this, BPA is considered an estrogen-mimicking type of endocrine disruptor that can deregulate endocrine signaling during the perinatal period, causing alterations in estrogen receptor numbers and behavioral pathways [25]. Among the systems altered by prenatal BPA exposure, there is evidence that the response to stress stimuli may be altered. Generally, an alteration in stress system regulation leads to changes in behavioral phenotypes related to anxiety and depression, in the same way as perinatal and childhood exposure to BPA. In particular, prenatal exposure to BPA, although the data are partly conflicting, induces anxiety in children, reporting also a sex differences, in which the relationship between early exposure to contaminants and anxiety symptoms in adolescence is observable only in boys [26]. This controversy is probably due to the weeks of exposure during gestation, suggesting a critical time window of contact [27]. In addition, for depressive symptoms, there seems to be a correlation with the exposure period and gender. In fact, gestational BPA appears to be associated with reactive behavior in boys, as measured by the Child Behavior Checklist, understood as a behavior characterized by a spontaneous emotion-driven response to perceived provocation [28].

Another important aspect to point out, based on evidence obtained in animal models, is that exposure to environmental contaminants (such as EDCs) during pregnancy, alters the mother’s behavior, leading to a decrease in engagement in maternal care, with transgenerational effect on maternal behavior, meaning that exposure to toxicants could also influence subsequent generations [29,30]. In line with this transgenerational effect, epigenetic changes play a key role in the effects on behavior, on cognitive functions, and on health-related alterations consequent to contaminant exposure, and seem to be involved in the Developmental Origins of Health and Disease (DOHaD) hypothesis [31]. According to the DOHaD hypothesis, diseases, health problems, and mortality during adulthood, originate in early life and are linked to experiences and exposure, also during intra-uterine environment [32]. Recently, in a large birth cohort study in Mexico, it was demonstrated that contaminant exposures have implications for child development through epigenetic changes; in particular, maternal bone lead burden impacted the developing fetus’ epigenome [33]. However, this area of research has a great limitation, because most studies on epigenetic role have been conducted on animals and do not include child developmental outcomes.

Moreover, for their normal daily activities, children drink more water, eat more food, and breathe more air compared to adults; especially, children during the first six months drink seven times as much water and children between ages 1 through 5 years eat 3 to 4 times more food with respect body weight. In addition, inhalation of air in standard conditions is twice that of adults, relative to body weight, and obviously entailing greater health risks.

Thus, analysis of the methods of exposure to environmental contaminants with consequent health effects at various periods of development is a crucial requirement to define a child-protective framework of risk assessment and stratification. In addition, we cannot forget that although genetic factors can contribute to non-communicable diseases, exacerbated also by combined influence of stress and other lifestyle habits, their rapid increase suggests that there may be other dynamics involved, probably linked to the environment, thus suggesting that it becomes necessary to identify, understand, and if possible prevent, variables that can alter the perinatal environment by increasing the susceptibility and the risk to develop pathologies in the long term.

## 3. Vulnerability to Contaminants: From Environmental to Lifestyle Chemical Agents

Of more than 80,000 chemical agents registered by the Environmental Protection Agency (EPA) for commercial use, only a small part is considered to have dangerous properties, and about 200 of these are classified as neurotoxic for humans. In this regard, data on effects in children are still scarce and controversial. Evidence in the epidemiological field showed a relationship between environmental contaminants, such as toxic metals, air pollution, and a variety of pesticides with adverse effects on brain development in children. Toxic metals, in particular, have a neurotoxic effect, considering that neurotoxicity is what alters structure and function of the central nervous system [34]. Examples of neurotoxicants include lead, mercury, and organophosphate (OP) pesticides. Until a few years ago, one of the main methods to identify whether a substance had neurotoxic effects was the assessment of high-dose exposure. Today, thanks to in vivo and in vitro cellular models, more sophisticated diagnostics tools have been developed to identify harmful effects, even at low doses. However, these studies, at the molecular level, cannot identify effects such as diseases and behavioral responses, therefore it is difficult to interpret whether they are actually harmful and to relate them to actual effects in a whole living organism.

Lead is an indicative example, in fact, although there has been a decline in its harmful effect since its removal from gasoline, exposure continues to occur in utero. Usually, in infancy, exposure can occur by means of ingestion or inhalation, but if high doses induce severe disabilities and sometimes even death, low doses can cause learning disabilities [35]. In fact, many studies have documented that a chronic low exposure to lead may decrease intelligence quotient (IQ), and cause attention deficit and behavioral problems [36]. For example, a study conducted among Inuit children in Northern Quebec, showed that childhood lead exposure is associated with externalizing behaviors and cannabis use during adolescence [37].

Furthermore, methyl mercury is another chemical agent with neurodevelopmentally adverse effects, in particular during the prenatal period in which it is transferred from maternal blood to the fetal brain and is excreted in breast milk. Mercury is deposited in lakes, oceans, seas, and therefore it accumulates in fish that, if ingested at high enough doses by pregnant women, may induce harmful effects in offspring, such as cerebral palsy and other neuropsychological impairments [38].

In children, non-communicable diseases, especially respiratory disorders, are also related to various air pollutants. A possible source includes wood stoves or materials that discharge organic gases and vapors with formaldehyde, resulting in symptoms that mimic influenza, such as fatigue, headache, dizziness, nausea and vomiting, cognitive impairment, and tachycardia [39]. In addition, the increase in recent years of asthma morbidity could be due to ambient air pollution. In fact, daily fluctuations in particulate matter (PM10) is responsible for a series of alterations, from an increased risk of asthma to autistic spectrum disorder [40,41]. In addition, epidemiological studies have shown that there is a relationship between ambient air pollutants, especially carbon monoxide (CO), and low birth weight, prematurity, and increased risk of congenital diseases [42]. Among the chemicals linked to lifestyle habits that can induce harmful effects in children, environmental tobacco smoke (ETS), considered as a mixture of substances created during the burning and smoking of tobacco, inducing effects that are partially found only in the fetal period. Elevated predisposition to low birth weight is an effect of prenatal exposure, and considering that low birth weight is associated with infant morbidity and mortality and chronic disease in adulthood, ETS can be responsible for enhancing the burden of disease [43]. In children, instead, ETS exposure involves an impairment of the upper and lower respiratory tract, in particular, when under 3 months of age, as well as lung growth and development [44]. This means that, unlike other environmental contaminants, an early ETS exposure may have fewer short-term effects, but harmful long-term effects, suggesting the need for longitudinal studies to understand countermeasures that need to be implemented. Recently, alongside traditional environmental contaminants, also those that are consequential in manufacturing activities, such as plastic pollution and electronic waste (e-waste), have become the fastest increasing waste stream worldwide, with probable harmful public health effects [45]. Although data in this field are still scarce, the timing and method of exposure to e-waste are closely related to the social and economic context. In developing countries, for example, such as India, Mexico, Ghana, and Nigeria, many e-waste activities involve child labor [46,47] and the health effects have been highlighted by higher concentrations of toxic metals in the blood of e-waste workers [46,47]. In particular, the exposure of pregnant women or children to e-waste causes neurodevelopmental disorders and fetal alterations [48,49]. Spontaneous abortions, stillbirths, premature births, and reduction in weight, height, and body-mass index have been reported [50].

## 4. The Risk Assessment Issue for Environmental Contaminants in Children

Protection of children’s health against the harmful consequences of exposure to environmental contaminants will need an approach that is not based exclusively on the consideration of average levels of exposure, but rather taking into account the critical periods of sensitivity and the impact on health in the short, medium, and long-term of that exposure.

Current methods include direct and indirect approaches, in order to estimate both the exposure and dose. Direct analysis is aimed at a short-term evaluation, such as air monitors or skin patches, while indirect approaches, based on algorithms and mathematical models, are more flexible in monitoring long-term effects.

If a direct approach usually considers an exposure typically over a shorter period of time, a modeling procedure uses information on activity patterns and environmental concentrations [51]. However, both criteria suffer from limitations related to a child’s activity that change with age, such as mouthing, sleeping, and eating, or are related to physiological patterns, such as heart rate and breathing rate, or are due to nutritional habits (water and juice intake, fruit consumption, meat consumption), and type of exposure (for example dermal) [51].

In addition, a model of risk assessment should consider multiple chemicals of different potency, through different exposure routes, in order to assess their cumulative effects. For this purpose, it might be useful to use a Monte Carlo model, which combines various datasets and permits examination of full exposure distributions. Thanks to the support of animal studies, new study approaches may be able to reliably detect the consequences of exposure during the prenatal and postnatal periods [52]. This will allow us to create databases on the effects on nervous, immune, respiratory, reproductive, cardiovascular and endocrine systems, not yet fully studied, especially in the long term. In addition, the physiological and biochemical responses of children at different stages of development, which influence metabolism during exposure to toxicants need to be considered through models that quantify the dose of toxic metabolites in different time windows. The EPA’s Child-Specific Exposure Factors Handbook and the EPA’s Child-Specific Exposure Scenarios Examples offer general child activity patterns and exposure factors are a useful first step in this direction [53,54].

In this context, as early as 1993, the National Research Council (NRC) requested the development of a risk assessment approach that takes into account the exposure of children and their specific susceptibility to pesticides [55]. The NRC report was based on the U.S. EPA developmental toxicity risk assessment, published in 1986 [53,54]. This suggests that children must be considered as a group in the population that needs special consideration in risk assessment. This report shows the key factors needed for the development of a preventive approach in risk assessment. First, there is a need to improve the quantitative assessment of children’s exposure at different stages of life, from fetal life to adolescence, considering acute, chronic, and multiple-pathway exposures. New approaches should be able to assess different functional, organ, cellular, and molecular outcomes, over the entire life span, also thanks to toxicodynamic and toxicokinetic methodologies.

## 5. Protective Factors to Reduce Effects of Contaminants Exposure: Current Strategies and Future Trends

Exposure to environmental contaminants does not necessarily involve negative developmental outcomes, thanks to several factors that could mitigate harmful effects. In particular, psychological, biological, and nutritional elements, due to individual differences, could result in protective reduction of health-related risk factors associated with exposure to contaminants [56]. One of these factors, studied mainly in animal models, is represented by “environmental enrichment”. One study has revealed that rats exposed to lead that lived in an enriched environment with additional space and toys in their cages, had fewer spatial learning deficits [57]. The idea that children living in impoverished environments are at greater risk of suffering dangerous effects from lead exposure has been around for decades; however, little attention has been given to the fact that an enriched environment might prevent or reduce these effects.

In general, the impact of environmental enrichment on children’s prognoses after toxicant exposure is indirect; children born in deprived settings are more likely, especially in the prenatal period, to be exposed to polycyclic aromatic hydrocarbons [58]. Alongside the role of environmental enrichment as a countermeasure, and probably correlated with it, there are nutritional factors. Diet may play a key role in mitigating the negative outcomes correlated with contaminant exposure. Animal models have demonstrated that lycopene, an antioxidant present in many foods, including tomato, may protect against the toxic effects of mercury, showing a lower levels of mercury in children who eat more tomato [59]. Other protective nutrients include iron and zinc, suggesting that iron supplementation may reduce the risk of lead poisoning and lower blood lead concentrations [60]. Some studies also revealed that children exposed to air pollution may have low levels of Vitamin D, therefore proposing their intake and supplementation [61].

However, while the nutritional factors are favorable sources of protection from risks of environmental contaminants, it is likely that there are other factors that are potentially involved, such as those of a psychosocial nature, although studies on this are still scarce. First of all, especially in the perinatal period and in early childhood, it is important to consider that exposure to toxic contaminants can compromise the functioning of parents within a familiar context. In this regard, a study on lead exposure in Mexico City revealed that maternal self-esteem predicted higher scores on the Bayley Scales of Infant Development (BSID), highlighting that maternal behavior reduced the negative association between blood lead levels and BSID scores in children and, thus, the parent–child relationship can mitigate the risks due to exposure to contaminants [62]. According to this perspective, it is important to consider the pathogenetic mechanisms of environment-induced diseases in children according to a multidisciplinary and integrated approach, which considers the possible effects at the functional, organ, cellular, and molecular levels. In line with this view, the PENSAMI Project “A precision medicine-based framework to pediatric patients with chronic diseases”, a prospective large-scale multicenter study that focuses on secondary prevention for pediatric patients with chronic diseases, is based on a perspective that an integrated and multidimensional health framework, including clinical, lifestyle, psychosocial, and environmental determinants, is effective in order to more accurately stratify patients with a higher probability of adverse health effects (clinical disease or death), and to improve the quality of life of pediatric patients with diabetes, asthma, and congenital cardiac diseases. This project stems from the need to help pediatric patients in the management of chronic diseases that must be managed throughout their life. In this context, the spectrum of risk factors ranges from purely genetic to behavioral, and psychosocial, creating an integrated and complex causal path which also embraces the environment, to be considered when applying preventive strategies to improve health. Many clinical and epidemiological studies focused on the association between environmental exposures and pediatric disease, but according the new directions in understanding the role of environmental contaminants in child outcomes, we cannot fail to consider those social, psychological and environmental dimensions, in a broad sense, strictly correlated, not only to risk factors, but also to preventive factors. Furthermore, according to a more general and integrated approach in the management of chronic diseases in pediatric patients, environmental data, as recognized by the WHO, have a significant clinical and quality of life weight, inducing acute and chronic conditions [63]. The relationship between environmental pollution and morbidity and mortality from neoplasms and respiratory diseases has been demonstrated in various epidemiological studies [64]. More recently, an association between environmental data and chronic degenerative diseases has been documented, including cardiovascular disease [65]. This study was conducted in a cohort of Italian adult subjects living in Tuscany. Nowadays, there are few data on the effects of environmental contaminants in Italian children or during pregnancy as predictors of a child’s future health outcomes [66,67].

Therefore, studies are needed to assess these potential effects. Therefore, it is reasonable to hypothesize that the variables belonging to clinical and psychosocial domains, associated with environmental measurements (dose, exposure period, toxicity, etc.) be included in a multidimensional and integrated framework for the challenging management of childhood development in order to improve clinical/educational/social decision making, reduce negative events, and improve quality of life.

## 6. Conclusions

**Figure 1:**
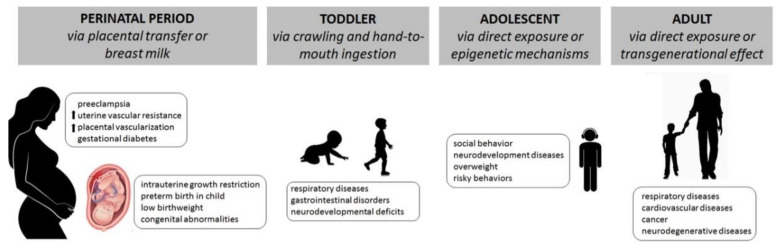
The effects of environmental hazards exposure across lifespan.

Environmental contaminants impair several dimensions of child development, with consequences on health status across lifespan, as described in Figure 1.

**Figure 2:**
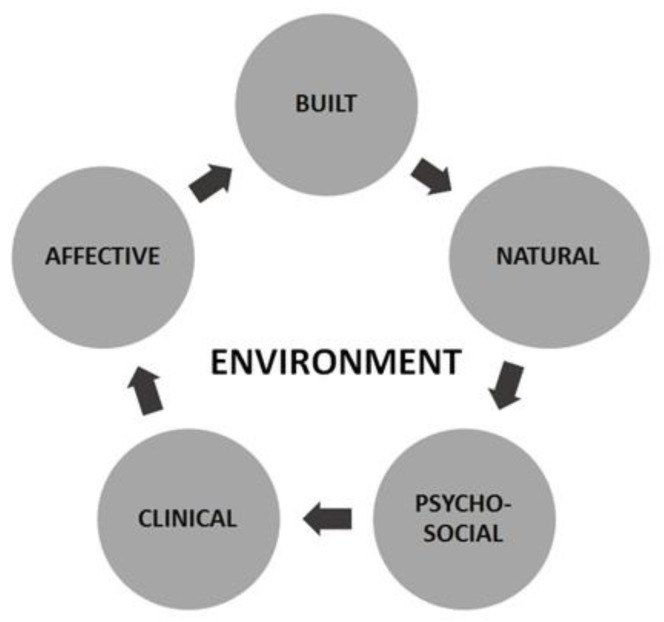
New framework of risk assessment and risk management.

Despite considerable progress being made toward understanding the role of chemicals in multiple domains of development, from embryonic and fetal periods to infancy, the need, in this context, is to design approaches to assess risk that account for clinical effects of contaminants, but are also centered on the impact that these may have on the socialization process, emotional health, and general activities of life. This especially true considering that the meaning of “environment” is rapidly changing; however, above all there is a greater realization of the intimate connections between the environment and human health, as depicted in Figure 2.

Taken as a whole, all evaluations suggest that risk factors belong to three broad areas: the built, natural, and social environments, accounting for the development of an integrated approach embracing the clinical and psychosocial perspectives, is the most effective way to prevent and control them. An integrated approach responds not only to the need for intervention in major common risk factors with the aim of reducing premature mortality and morbidity of chronic diseases in childhood and later in life, but also has to integrate primary, secondary and tertiary prevention, health promotion, and related programs across sectors and different disciplines.
